# Evaluation of Candida bloodstream infection and antifungal utilization in a tertiary care hospital

**DOI:** 10.1186/s12879-018-3094-9

**Published:** 2018-04-18

**Authors:** Tatiana Aporta Marins, Alexandre R. Marra, Michael B. Edmond, Marines Dalla Valle Martino, Paula Kiyomi Onaga Yokota, Ana Carolina Cintra Nunes Mafra, Marcelino Souza Durão Junior

**Affiliations:** 1 Hospital Israelita Albert Einstein, São Paulo, Brazil; 2 Division of Medical Practice, Hospital Israelita Albert Einstein, São Paulo, Brazil; 3 Office of Clinical Quality, Safety and Performance Improvement, University of Iowa Hospitals and Clinics, Iowa City, IA USA; 4 Division of Infectious Diseases, Department of Internal Medicine, University of Iowa Carver College of Medicine, Iowa City, IA USA; 5 Clinical Laboratory, Hospital Israelita Albert Einstein, São Paulo, Brazil; 6 Instituto Israelita de Ensino e Pesquisa Albert Einstein, Hospital Israelita Albert Einstein, São Paulo, Brazil

**Keywords:** Antifungal, Candidemia, Consumption, Defined daily dose, Days of therapy

## Abstract

**Background:**

Candida bloodstream infections carry a significant mortality risk, justifying the importance of adequate antifungal therapy. This study describes trends in antifungal consumption using the Defined Daily Dose (DDD) and Days of Therapy (DOT) metrics, identifies the microbiological profile, the time to initiation of empirical therapy, the adjustment after positive blood culture results for *Candida*, and the impact on in-hospital mortality rate in patients with candidemia.

**Methods:**

An analysis of antifungal consumption from 2008 to 2016, and of candidemia cases from 2012 to 2016 was carried out in a private tertiary hospital.

**Results:**

A total of 11,273 admissions were identified with a prescription for at least one type of antifungal therapy. Fluconazole was the most prescribed antifungal drug in terms of general consumption. Through the DDD and DOT metrics, we observed that over time, there was an increase in the consumption of liposomal amphotericin B, micafungin and voriconazole. *Candida albicans* was the most isolated species in blood cultures. Regarding candidemia, we analyzed samples from 115 patients. Empirical therapy was started within 24 h of blood culture in 44.3% of the cases, and in 81.7% of the cases, the antifungal was deemed to be adequate based in antifungal susceptibility testing, both of which were not associated with the in-hospital mortality rate.

**Conclusions:**

Our study reinforces the importance of monitoring the consumption of antifungal agents, which helps in proposing actions that lead to their rational use and, consequently, reduces the appearance of resistant strains.

## Background

Since the 1980s, candidemia cases have increased substantially in different regions of the world and, in Brazilian hospitals, it is one of the highest incidence infections [1, 2]. In a recently published Brazilian study, *Candida spp.* was the seventh most prevalent agent in analyzed bloodstream infections [3]. *Candida albicans* remains the most commonly isolated microorganism among *Candida*, however, candidemia caused by non*-albicans* species has increased worldwide, particularly *C. tropicalis*, *C. glabrata*, *C. parapsilosis* and *C. krusei* [4, 5].

The severity of these infections poses a significant morbidity and mortality risk [6]. They are often expensive and difficult to treat, so adequate antifungal therapy is necessary [7–10]. Regarding the timing of initiation of empiric treatment, studies are still controversial, with some showing that mortality was lower in patients who received the antifungal early [10, 11] and others indicating that there was no significant difference in in-hospital mortality [12, 13].

In order to provide adequate antifungal therapy, it is important to know the consumption pattern and the microbiological profile of your institution and, with the obtained information, to implement opportunities for improvement that lead to the rational use of these drugs and consequently achieve therapeutic success and increase patient safety.

Despite the advances in antifungal development in the previous decade, the therapeutic arsenal available on the market, and validated for the treatment of systemic fungal infections, is still limited and candidemia and other forms of invasive candidiasis remain potentially fatal infections, justifying the careful choice of treatments [14, 15]. Currently, there are four classes of systemic antifungal drugs that are effective: polyenes, azoles, echinocandins, and flucytosine [16]. Consumption of these drugs should be monitored over the years to control overuse, measure improvements, and obtain parameters for future internal and external comparisons. Monitoring is also important to prevent resistant microorganism species in the future.

This monitoring should be performed through quantitative analysis [17, 18]. There are already well established metrics for the consumption of antimicrobials, the most common being the Defined Daily Dose (DDD), but there are others such as: days of therapy (DOT), Prescribed Daily Dose (PDD), and Recommended Daily Dose (RDD), which are also being used for the calculation of antifungal consumption [19–22]. There is still no consensus as to what is the best metric to quantify the use of these drugs. The World Health Organization (WHO) recommends consumption calculation using DDD [22], but other authors suggest DOT, since this methodology is not influenced by changes in the recommended individual dose and by dose adjustment in cases of kidney failure [23, 24].

The goal of this study is to describe and analyze antifungal consumption trends through the DDD and DOT metrics in patients who were admitted to Hospital Israelita Albert Einstein, and also identify the microbiological profile, time of onset of empirical antifungal therapy, antifungal adequacy and the impact on in-hospital mortality in patients with candidemia.

## Methods

The study was conducted in a 670-bed private, tertiary hospital focused on high complexity treatments including solid organ and bone marrow transplantation, located in the city of São Paulo, Brazil. This is a descriptive and retrospective observational study that identified the global consumption of antifungal drugs in all the patients who were hospitalized from January 1, 2008 to December 31, 2016. Adult inpatients with candidemia from January 1, 2012 to December 31, 2016 were also analyzed. This study was submitted and approved by the Institution’s Research Ethics Committee and informed consent was not required.

The report of inpatient antifungal medicines was generated through the hospital’s electronic inventory management system. The analyzed drugs were: fluconazole, voriconazole, itraconazole, posaconazole, caspofungin, micafungin, anidulafungin, liposomal amphotericin B and amphotericin B deoxycholate. Hospital nursing units include Adult Medical and Surgical Wards (MSW, *n =* 14), Oncology (ONCO), Bone Marrow Transplantation (BMT), Solid Organ Transplantation (SOT), Adult Intensive Care Unit (AICU), Pediatrics (PED) and Neonatal Intensive Care Unit (NICU).

The impact of bed occupancy variation was minimized by reporting the antifungal consumption per 1000 patient-days. Two consumption metrics were used, DDD and DOT. We chose these metrics because they are the most commonly used and are recommended by health agencies [20, 22].

DDD is defined as the mean maintenance dose per day for a drug used in its primary indication in an adult patient [22]. The DOT metric measures the number of days that a patient is on a specific drug, regardless of the number of doses administered or dosing [25]. One DOT is assigned for each drug administered each day. To assess consumption in Pediatrics and Neonatology, only the DOT was calculated. DDD and DOT were calculated annually for general consumption data, and data by specialty. The formulas used for the DDD and DOT calculations were:

$$ \mathrm{DDD}\;1000\;\mathrm{patient}\hbox{-} \mathrm{days}=\frac{\mathrm{drug}\kern0.17em \mathrm{consumption}\kern0.17em \mathrm{in}\kern0.17em \mathrm{grams}\kern0.17em \mathrm{for}\kern0.17em \mathrm{period}\times 1000}{\mathrm{IDD}\times \mathrm{number}\kern0.17em \mathrm{of}\kern0.17em \mathrm{patient}\hbox{-} \mathrm{days}\kern0.17em \mathrm{in}\kern0.17em \mathrm{the}\kern0.17em \mathrm{period}} $$

$$ \mathrm{DOT}\;1000\;\mathrm{patient}\hbox{-} \mathrm{days}=\frac{\mathrm{days}\kern0.17em \mathrm{of}\kern0.17em \mathrm{the}\mathrm{rapy}\kern0.17em \mathrm{in}\kern0.17em \mathrm{the}\kern0.17em \mathrm{period}\times 1000}{\mathrm{number}\kern0.17em \mathrm{of}\kern0.17em \mathrm{patient}\hbox{-} \mathrm{days}\kern0.17em \mathrm{in}\kern0.17em \mathrm{the}\kern0.17em \mathrm{period}} $$

For the DDD calculation, the anatomical therapeutic chemical (ATC)/DDD system, recognized by the World Health Organization as an international standard for drug use studies, was used. The Defined Daily Doses used were 200 mg for fluconazole, 400 mg for voriconazole, 200 mg for itraconazole, 300 mg for posaconazole, 50 mg for caspofungin, 100 mg for micafungin, 100 mg for anidulafungin and 35 mg for amphotericin B deoxycholate and liposomal amphotericin B [22].

In analyzing the medical records for hospitalized patients diagnosed with candidemia in the period between 2012 and 2016, patients under the age of 18 years, outpatients and those who had incomplete data in their medical records were excluded. The data gathered followed a standard form including the following variables: age, gender, admission weight, admission date, hospitalization time, antifungal used in the treatment, dose, time of treatment, date of collection and date of blood culture result, type of microbiological agent, antifungal sensitivity test, time of starting empiric therapy, adequacy based on antifungal susceptibility testing, prescribed dose as recommended by the institutional protocol for invasive fungal infections, and in-hospital outcome.

Patients’ primary comorbidities were identified, as well as the presence of risk factors for candidemia (intensive care unit admission, mechanical ventilation, hemodialysis, blood transfusion, neutropenia (total neutrophil count < 500/mm^3^), use of vasoactive drugs, antimicrobial agents, corticosteroids, chemotherapy drugs, parenteral nutrition, surgical procedure, presence of central venous catheter). *Breakthrough* cases were also identified, defined as a positive blood culture during antifungal therapy of at least three days duration. These *breakthrough* cases were also included in our analysis [26, 27].

A candidemia episode was defined as the isolation of *Candida* in blood culture. For all patients, the first episode of candidemia was the episode analyzed. Polymicrobial blood cultures, that are where there was growth of other microorganisms in addition to *Candida* species, were excluded from the analysis.

Empirical therapy was defined as the initiation of an antifungal drug without knowledge of the infection-causing microorganism species, and targeted therapy was defined as treatment with an antifungal drug after the culture result was reported.

Empirical therapy was considered inadequate when no intravenous antifungal agent was administered within 24 h of blood culture [11, 28]. After obtaining the blood culture result identifying the microorganism and the antifungal susceptibility testing, therapy was considered inadequate if the isolated organism was resistant to the antifungal agent in use (for example, fluconazole for *Candida krusei*), if the antifungal agent was used in inadequate doses (e.g. fluconazole doses of less than 400 mg per day) or if no antifungal agent was initiated [10, 11, 29].

The hospital has an institutional protocol to guide the clinical staff based on international antifungal guidelines. Doses recommended by the institutional protocol were as follows: liposomal amphotericin B 3 to 5 mg/kg/day, fluconazole 400 to 800 mg/day orally or intravenously, voriconazole 6 mg/kg IV every 12 h on day 1 followed by 4 mg/kg/day intravenously or orally, caspofungin loading dose of 70 mg followed by 50 mg/day thereafter, anidulafungin 200 mg loading dose followed by 100 mg/day thereafter, and micafungin 100 mg/day [16, 30].

### Microbiological analysis

All positive samples were identified using BACTEC FX equipment (BD Diagnostic Systems, Franklin Lakes, NJ, USA), an automated method used to detect micro-organisms in blood cultures. After yeast were identified by gram stain, the chromogenic agar ChromID CPS (bioMérieux) was used, allowing identification of yeasts by the colors produced in the medium. Until 2013, species identification was carried out by VITEK® 2 (bioMérieux) and from then on, to the present date, mass spectrometry VITEK MS (bioMérieux, Marcy l’Etoile, France) and Maldi-TOF (Bruker Microflex LT Biotyper 3.1) was used.

The antifungal susceptibility test profile was carried out for all blood culture samples positive for *Candida,* using the broth microdilution method according to CLSI document M27-A3, using current CLSI MIC interpretative criteria as M27-S4 [31, 32]. *Candida parapsilosis* ATCC22019 and *Candida krusei* ATCC6258 were used as quality controls. Commonly tested drugs were amphotericin B, fluconazole, voriconazole, anidulafungin (or caspofungin, depending on laboratory availability). Micafungin susceptibility testing was not performed because the laboratory did not have the specific test supplies for this drug during the study period.

### Statistical analysis

In order to compare the DDD and DOT measurements, and to assess possible trends over time, Spearman correlation coefficients were used. In the comparison between hospitalization units in terms of antifungal consumption metrics, Kruskal-Wallis hypothesis tests were used, with post-tests corrected by the Steel [33] method, considering the medical and surgical clinic as a reference category.

Anidulafungin was introduced in the hospital formulary in 2010, micafungin in 2011 and posaconazole in 2016. These periods without consumption of these drugs were considered missing in the analysis. For the analysis of variations over time and for comparisons between units, if a unit had not consumed a certain antifungal in any valid year, the DDD and DOT values were considered zero. For correlations between DDD and DOT, zero values were not considered, but only the values or the years in which there was some antifungal consumption.

In order to investigate the in-hospital mortality associated factors, simple and multiple logistic regression models were fitted. For the multiple model, a stepwise variable selection process was conducted in both directions, considering information on the patient’s profile, clinical history, antifungal treatment and hospitalization. The criterion for selection of variables was Akaike information (AIC). Model results are represented by *p* values, odds ratios (OR) and their 95% confidence intervals (95% CI). The analyses were performed using R 3.1.3 (R Core Team, 2015). The significance level adopted was 5%.

## Results

**Fig. 1:**
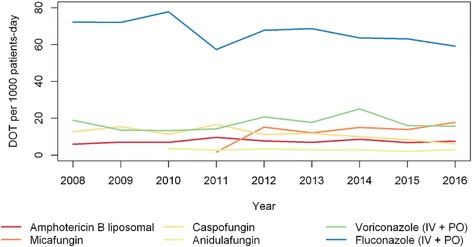
Days of Therapy (DOT) per 1000 patient-days from 2008 to 2016. IV: intravenous; PO: oral

We found high correlation between the DDD and the study period indicating that there was a significant increase in the consumption of amphotericin B (rho_DDD_ = 0.87) over time, particularly the liposomal formulation (rho_DDD_ = 0.83), micafungin (rho_DDD_ = 0.89), and intravenous voriconazole (rho_DDD_ = 0.80). There was a significant reduction in the consumption of caspofungin (rho_DDD_ = − 0.80) and intravenous fluconazole (rho_DDD_ = − 0.88) over time. The temporal DOT trends followed along the same DDD lines, but with some differences in intensity and significance. Figure 1 shows the antifungal consumption using DOT throughout the study period from 2008 to 2016.

In investigating the correlation between DDD and DOT, the values of the correlation coefficients indicated positive correlations: the higher the DDD, the higher the DOT, but the correlation was not significant for all drugs.

Table 1 shows there was a significant difference between the utilization of all antifungal agents studied in the different hospitalization units, both according to DDD and DOT measurements. The consumption of posaconazole was not analyzed because it had only one measure in 2016.

**Table 1:** Median antifungal consumption in hospital units between 2008 and 2016

Drug\Unit	MSC	Onco	Ped	AICU	BMT	TX	NICU	*P* Value*
DDD per 1000 patient-days								
Amphotericin B (lipos + deox)	14.2	190.9		239.4	359.0	79.2		< 0.001
*P* Value		0.002		0.002	0.002	0.045		
Amphotericin B liposomal	13.8	190.9		237.5	351.3	78.9		< 0.001
*P* Value		0.002		0.002	0.002	0.110		
Amphotericin B deoxycholate	0.4	0.0		3.2	0.0	7.9		0.012
*P* Value		0.904		0.110	0.940	0.135		
Anidulafugin (2010–2016)	1.4	11.6		20.0	6.2	0.0		0.002
*P* Value		0.009		0.009	0.995	0.568		
Caspofungin	7.2	28.1		73.3	46.7	2.0		< 0.001
*P* Value		0.007		0.002	0.057	0.110		
Fluconazole (IV + PO)	35.7	257.9		160.7	625.6	159.2		< 0.001
*P* Value		0.002		0.002	0.002	0.002		
Fluconazole L IV	25.3	163.3		150.7	382.3	52.6		< 0.001
*P* Value		0.002		0.002	0.002	0.002		
Fluconazole PO	11.4	124.9		14.3	134.2	102.8		< 0.001
*P* Value		0.002		0.035	0.002	0.002		
Itraconazole	0.7	2.7		0.6	0.00	14.7		0.001
*P* Value		0.625		0.690	0.006	0.324		
Micafungina (2011–2016)	3.7	41.8		71.7	153.6	21.2		0.001
*P* Value		0.020		0.077	0.020	0.224		
Posaconazole (2016)	0.0	44.4		6.9	87.3	0.0		–
Voriconazole (IV + PO)	3.1	82.6		22.2	174.4	7.1		< 0.001
*P* Value		0.002		0.002	0.002	0.495		
Voriconazole IV	1.7	44.4		16.0	83.0	3.7		< 0.001
*P* Value		0.002		0.002	0.002	0.993		
Voriconazole PO	1.4	42.4		5.4	80.6	3.4		< 0.001
*P* Value		0.002		0.007	0.002	0.324		
DOT per 1000 patient-days								
Amphotericin B (lipos + deox)	3.2	29.8	20.2	38.9	85.2	16.0	2.6	< 0.001
*P* Value		0.002	0.016	0.002	0.002	0.016	0.943	
Amphotericin B liposomal	2.5	29.8	19.7	36.0	82.8	10.8	0.5	< 0.001
*P* Value		0.002	0.004	0.002	0.002	0.134	0.069	
Amphotericin B deoxycholate	0.4	0.0	0.0	1.6	0.0	5.2	1.1	< 0.001
*P* Value		0.745	0.100	0.045	0.966	0.028	0.434	
Anidulafugin (2010–2016)	1.3	11.2	0.0	18.4	5.4	0.0	0.0	< 0.001
*P* Value		0.009	0.377	0.009	0.995	0.568	0.005	
Caspofungin	7.0	27.0	15.0	65.4	45.9	1.8	0.0	< 0.001
*P* Value		0.007	0.057	0.002	0.089	0.057	0.001	
Fluconazole (IV + PO)	42.9	237.0	41.5	147.7	420.1	174.4	1.1	< 0.001
*P* Value		0.002	0.984	0.002	0.002	0.002	0.002	
Fluconazole L IV	23.4	102.0	29.8	126.7	259.4	51.1	1.1	< 0.001
*P* Value		0.002	0.690	0.002	0.002	0.002	0.002	
Fluconazole PO	20.1	130.4	13.2	23.6	160.6	119.7	0.0	< 0.001
*P* Value		0.002	0.057	0.434	0.002	0.002	0.001	
Itraconazole	0.8	1.2	1.0	0.8	0.0	11.8	0.0	< 0.001
*P* Value		0.753	1.000	0.812	0.008	0.324	0.001	
Micafungina (2011–2016)	3.9	45.3	8.5	73.5	225.5	21.8	0.0	< 0.001
*P* Value		0.020	0.624	0.077	0.020	0.224	0.024	
Posaconazole (2016)	0.0	3.1	0.0	0.5	6.2	0.0	0.0	–
Voriconazole (IV + PO)	5.4	130.1	35.0	25.4	322.3	10.1	0.00	< 0.001
*P* Value		0.002	0.002	0.002	0.002	0.559	0.001	
Voriconazole IV	2.6	57.2	14.0	20.1	142.7	4.7	0.00	< 0.001
*P* Value		0.002	0.002	0.002	0.002	0.984	0.001	
Voriconazole PO	2.2	73.7	17.7	7.1	147.7	5.6	0.00	< 0.001
*P* Value		0.002	0.028	0.002	0.002	0.276	0.001	

*DDD* Defined Daily Dose, *DOT* Days of Therapy, *IV* Intravenous, *PO* Oral, *MSW* Medical and Surgical Wards, *Onco* Oncology, *Ped* Pediatrics, *AICU* Adult Intensive Care Unit, *BTM* Bone marrow transplant, *SOT* Solid organ transplant, *NICU* Neonatal Intensive Care Unit *P values for Kruskal-Wallis tests to test whether in at least one of the units the consumption was different from the others. Other *p*-values for post-tests with Steel correction that compare each unit with the Medical and Surgical Wards

The most commonly used antifungal drug was fluconazole, followed by liposomal amphotericin B, and the units with the highest consumption of these drugs were the Bone Marrow Transplantation, Oncology, Adult Intensive Care, and Solid Organ Transplantation Units (Table 1).

In the period between 2008 and 2016 there were 11,273 hospitalizations with at least one antifungal prescribed to 6779 patients who had one to 25 admissions within the study period. In 64.9% of admissions with antifungal prescriptions, a blood culture test was requested, which was positive for *Candida* in 3.2% of cases.

One hundred fifteen patients developed candidemia between 2012 and 2016. The majority (61.7%) were men and the mean age was 68 years (SD: 17.9). Half of the patients died (51.3%) and mean length of stay of 36 days. Table 2 also shows the risk factors associated with candidemia present during hospitalization and the clinical outcomes.

**Table 2:** Hospital admission data and risk factors for patients with candidemia (*N* = 115)

Variables	Descriptions
Sex *n*(%)	
Female	44 (38.3)
Male	71 (61.7)
Age (years)	
Mean (SD)	68 (17.9)
Median (1st quartile - 3rd quartile)	70 [56–84]
Minimum maximum	26–100
Length of stay (days)	
Mean (SD)	69.8 (138.4)
Median (1st quartile - 3rd quartile)	36 [21–62.5]
Minimum maximum	1–1003
Use of antimicrobial	113 (98.3)
Use of chemotherapy drugs	12 (10.4)
Use of corticosteroids	95 (82.6)
Use of parenteral nutrition	46 (40.0)
Presence of some type of central venous catheter	86 (74.8)
Had any type of surgery	79 (68.7)
Admission to the intensive care unit	94 (81.7)
Length of ICU stay (days) (*n* = 94)	
Mean (SD)	23.3 (23.3)
Median (1st quartile - 3rd quartile)	15 (6–32)
Minimum maximum	1–100
Neutropenia	6 (5.2)
Use of vasoactive drug	86 (74.8)
Use of mechanical ventilation	82 (71.3)
Hemodialysis	38 (33.0)
Had hemotherapy	27 (23.5)
Positive blood culture even when using antifungal therapy	35 (30.4)
Clinical outcome	
Discharge from hospital	56 (48.7)
In-hospital mortality	59 (51.3)

Category variables presented by absolute and relative frequencies. *SD* standard deviation

In 13.9% (16/115) of patients with candidemia antifungals were not used. Eight of these cases died before blood cultures were obtained, four were discharged from the hospital before the results of blood culture returned (two of which were readmitted), and for the others we did not find reasons in the patient’s records for not treating the candidemia.

The duration of treatment with antifungals ranged from less than a full day to 149 days with a median of 14 days. The most commonly used antifungal agents were fluconazole (42.6%, 49/115), followed by micafungin (35.7%, 41/115) and caspofungin (24.3%, 28/115). The most heavily used class of antifungals was echinocandins (62.6%, 72/115). Each patient may have consumed more than one type of antifungal; there were cases of antifungal substitution during the same treatment, and in one patient there was a combination of two antifungals administered at the same time without justification in the medical record. As a result, 28.7% (33/115) of the patients received echinocandins only, 19.1% (22/115) azoles only and 4.3% (5/115) liposomal amphotericin B only. The *Candida* species most prevalent in blood cultures was *C. albicans* (36.5%, 42/115), followed by *C. parapsilosis* (22.6%, 26/115), *C. glabrata* (21.7%, 25/115), *C. tropicalis* (11.3%, 13/115), *C. krusei* (4.3%, 5/115), *C. guilliermondii* (1.7% /115). Two only species accounted for one case each.

Empiric therapy with antifungal drugs after blood cultures were drawn was started within 24 h in only 44.3% of the cases (Table 3). In 81.7% of cases (94/115), the empiric treatment was determined to be adequate after antifungal susceptibility tests were reviewed. The antifungal agent was changed in 36.5% of the cases. The first choice, in general, was fluconazole (44.4%, 44/99), caspofungin (22.2%, 22/99) or micafungin (22.2%, 22/99), and the most commonly used antifungal after switching was micafungin (17.4%, 20/115), followed by liposomal amphotericin B (13%, 15/115). Table 3 also shows cases where the dose was adequate according to institutional protocol.

**Table 3:** Empiric therapy in patients with candidemia (*N* = 115)

Variables	*n*(%)
Time to start empiric therapy with antifungal after blood culture obtained (hours)	
24	51 (44.3)
48	20 (17.4)
72	16 (13.9)
96	7 (6.1)
120	5 (4.3)
No use of antifungal drug	16 (13.9)
Appropriate empirical antifungal treatment according to antifungal susceptibility test result	
No	21 (18.3)
Yes	94 (81.7)
Adequate dose according to institutional protocol^a^	
Caspofungin	28/28 (100.0)
Micafungin	31/41 (75.6)
Anidulafungin	5/8 (62.5)
Fluconazole	12/49 (24.5)
Amphotericin B liposomal	20/21 (95.2)
Voriconazole	4/5 (80.0)

Category variables presented by absolute and relative frequencies

^a^number of cases observed/number of cases using antifungal drug

As for the clinical history, 59.1% (68/115) of patients suffered from cardiac disease, 27.8% (32/115) from diabetes mellitus, 23.5% (27/115) from nephropathy, 22.6% (26/115) from cancer, and 19.1% (22/115) were bone marrow and solid organ transplant patients.

In Table 4 are the variables that were evaluated by simple logistic regression analysis. In Table 5, after the construction of multiple logistic regression analysis, the factors that remained significant and associated with death risk were: age, length of stay in the ICU, use of vasoactive drugs, use of chemotherapy, hemodialysis and positive blood culture while on antifungal therapy (breakthrough candidemia).

**Table 4:** Data for hospitalization and risk factors, by type of discharge (*N* = 115)

	Discharge (*N* = 56)	In-hospital mortality (*N* = 59)	OR (95% CI)	*P* value
Gender				
Female	23 (41.1)	21 (35.6)	1.00	
Male	33 (58.9)	38 (64.4)	1.26 (0.59; 2.69)	0.546
Age (years)			1.03 (1.01; 1.05)	0.015
Mean (SD)	63.80 (19.33)	72.03 (15.53)		
Median [1st Q - 3rd Q]	65.00 [50–81.50]	76.00 [60–85]		
Length of stay (days)			1.00 (1.00; 1.00)	0.952
Mean (SD)	69.02 (140.67)	70.58 (137.32)		
Median [1st Q - 3rd Q]	32 [20.25–64.25]	36 [23.50–56]		
Time to start empiric therapy with antifungal after blood culture				
Within 24 h	23 (41.1)	28 (47.5)	1.00	
More than 24 h	27 (48.2)	21 (35.6)	0.64 (0.29; 1.41)	0.268
No use of antifungal drug	6 (10.7)	10 (16.9)	1.37 (0.44; 4.56)	0.593
Used antimicrobial				
No	1 (1.8)	1 (1.7)	1.00	
Yes	55 (98.2)	58 (98.3)	- 11-22-2016 17:19:0 s2	0.970
Used chemotherapy				
No	52 (92.9)	51 (86.4)	1.00	
Yes	4 (7.1)	8 (13.6)	2.04 (0.60; 8.03)	0.268
Used corticosteroid				
No	15 (26.8)	5 (8.5)	1.00	
Yes	41 (73.2)	54 (91.5)	3.95 (1.40; 12.96)	0.014
Used parenteral nutrition				
No	37 (66.1)	32 (54.2)	1.00	
Yes	19 (33.9)	27 (45.8)	1.64 (0.78; 3.53)	0.197
Used some type of central venous catheter				
No	22 (39.3)	7 (11.9)	1.00	
Yes	34 (60.7)	52 (88.1)	4.81 (1.93; 13.30)	0.001
Had any type of surgery				
No	20 (35.7)	16 (27.1)	1.00	
Yes	36 (64.3)	43 (72.9)	1.49 (0.68; 3.33)	0.322
Stayed at the intensive care unit				
No	17 (30.4)	4 (6.8)	1.00	
Yes	39 (69.6)	55 (93.2)	5.99 (2.04; 22.07)	0.003
Length of stay in the intensive care unit (days) (*n* = 94)			1.04 (1.02; 1.06)	0.001
Mean (SD)	16.03 (16.86)	28.42 (25.86)		
Median [1st Q - 3rd Q]	10.00 (5.00–17.50)	23.00 (7.50–36.00)		
Neutropenia				
No	54 (96.4)	55 (93.2)	1.00	
Yes	2 (3.6)	4 (6.8)	1.96 (0.37; 14.59)	0.447
Used vasoactive drug				
No	23 (41.1)	6 (10.2)	1.00	
Yes	33 (58.9)	53 (89.8)	6.16 (2.39; 18.12)	< 0.001
Used mechanical ventilation				
No	26 (46.4)	7 (11.9)	1.00	
Yes	30 (53.6)	52 (88.1)	6.44 (2.61; 17.74)	< 0.001
Hemodialysis				
No	45 (80.4)	32 (54.2)	1.00	
Yes	11 (19.6)	27 (45.8)	3.45 (1.53; 8.21)	0.004
Blood transfusion				
No	48 (85.7)	40 (67.8)	1.00	
Yes	8 (14.3)	19 (32.2)	2.85 (1.16; 7.55)	0.027
Positive blood culture even when using antifungal therapy				
No	44 (78.6)	36 (61.0)	1.00	
Yes	12 (21.4)	23 (39.0)	2.34 (1.04; 5.48)	0.043

Category variables presented by absolute and relative frequencies. *Q* quartile *SD* standard deviation, *OR* odds ratio obtained by simple logistic regression model (values, 1.00 for the reference categories) and, in parentheses, the 95% Confidence Interval (95% CI)

**Table 5:** Factors associated with death risk in patients with candidemia (N = 115)

Factors	OR (95% CI)	*P* value
Age (years)	1.04 (1.01; 1.08)	0.005
Treatment with antifungal (days)	0.95 (0.91; 0.99)	0.022
Time in ICU (days)	1.03 (1.01; 1.07)	0.020
Use of vasoactive drugs (Yes)	3.90 (1.18; 14.43)	0.031
Use of chemotherapy (Yes)	6.88 (1.37; 40.17)	0.023
Hemodialysis (Yes)	3.19 (1.11; 9.86)	0.035
Positive blood culture even when using antifungal drug (Yes)	4.20 (1.25; 15.86)	0.026

In parentheses, the 95% Confidence Interval (95% CI). *OR* odds ratio obtained by multiple logistic regression model

## Discussion

Analyzing the data, it was possible to identify a wide use of antifungal drugs, which may be related to the characteristics of the hospital, because it is large tertiary care facility This study is similar to a study carried out in Spain, where antifungal consumption was higher in large university hospitals [34].

In our study we observed a significant increase over time in the consumption of liposomal amphotericin B, micafungin and voriconazole, and a significant reduction in the consumption of caspofungin and fluconazole (Fig. 1). The increased use of liposomal amphotericin B and voriconazole is probably related to the empirical and prophylactic treatments used in the onco-hematology unit, and the increase of micafungin is due to its lower cost. We also suspect that the reduction of fluconazole usage may be related to the predominance of *C.* non-*albicans* species in our institution, and the reduction in the use of caspofungin due to the higher cost of this drug and the standardization of use of micafungin, which has lower cost and equivalent effectiveness. We believe that introducing an agent of the same class at a lower cost into the hospital formulary has caused this change in the pattern of echinocandin prescriptions in our institution over the years. Since studies do not show significant differences in efficacy among these drugs, cost may become a differential point in decision making over which drug to choose. Our findings were different from the Spanish study, where there was an increase in fluconazole consumption, followed by a smaller scale increase in voriconazole, and a reduction in itraconazole consumption [34]. In the same study, the consumption of echinocandins was more apparent in the ICU; we also observed in our study that the adult ICU had the highest consumption of caspofungin and anidulafungin (Table 1). In another study conducted in ICUs, a decrease in the use of fluconazole and an increase in echinocandin consumption also became evident over time [35]. In a Brazilian university hospital, the highest consumption was of the azole class, first fluconazole, followed by voriconazole and the third most consumed was amphotericin B deoxycholate, a fact that can be explained by the limitations in resources, since it is a public hospital, and this deoxycholate formulation is cheaper, with greater control over the use of liposomal amphotericin B and echinocandins, requiring authorization prior to the prescription of these high-cost drugs [36].

Two other German studies used different metrics, one Recommended Daily Dose (RDD) and the other Prescribed Daily Dose (PDD), and both showed a higher consumption of antifungal drugs in the ICU and Hematology/Oncology service [37, 38]. Our study shows that these units also have a high consumption of antifungal agents (Table 1), since antifungal prophylaxis is included in the treatment profile of these patients and the use of empiric therapy is quite common [38, 39].

When comparing our work with an earlier study performed at the same institution [1], where the DDD of antifungal drugs was calculated from 1997 to 2007, we can observe that, in general, there was an increase in the consumption of these drugs, but there was also an increase in the therapeutic arsenal available in our formulary. In 2010, anidulafungin was added, micafungin was added in 2011, and posaconazole in 2016. Fluconazole consumption remained stable, mean use 61.5 (range from 32.7 to 101.2) DDD/1000 patient-days in the first study, and mean use 62.14 (range from 50.11 to 70.66) DDD/1000 patient-days in the second study. Observation demonstrated that the use of amphotericin B deoxycholate continued to be reduced in our study, as in the previous one, due to the higher risk of nephrotoxicity associated with this formulation, which led to an increase in the prescription of liposomal amphotericin B and echinocandins.

For most drugs there was a positive correlation between DDD and DOT values, but not all of them were strong and significant. A different pattern was observed for amphotericins, which we believe to be due to the fact that when the dose administered exceeds the Individualized Daily Dose (IDD), the DDD significantly exceeds the DOT. For drugs such as caspofungin and micafungin, there is usually no significant difference between DDD and DOT per 1000 patient-days, as the daily administered dose is close to the IDD equivalent [23].

The ideal metric to measure drug consumption is still not well defined in the literature. The two metrics used in this study have both advantages and disadvantages, and most studies involve the consumption of antibacterial drugs. A primary concern with this class of drugs is bacterial resistance, which is increasing while the development of new agents is slow. The emergence of antifungal resistance should prompt the monitoring of utilization of these drugs.

DDD allows the comparison of utilization in different hospitals, regions and countries. It can be calculated even when a computerized pharmacy data system is not available, although electronic systems aids in optimizing the calculation time by the pharmacist in both metrics [23, 25]. Disadvantages of DDD are its inaccuracy in drugs that require dose adjustment for renal function or weight-based drugs. IDD values may change with the approval of new doses for existing drugs, which can create confusion when comparisons of use are made over time [23–25, 40]. The advantages of the DOT metric are greater accuracy than DDD, it can be used to measure utilization in children (weight-based regimens), is not influenced in case of changes in recommended doses of IDD, is not influenced by discrepancies between IDD and preferred daily doses or usual daily doses, which may vary between hospitals [23, 38]. The disadvantages of DOT are: calculation is more difficult without computerized systems and there is a lack of accuracy in populations with renal failure, although the values obtained with DOT calculation are more accurate than when calculating DDD in this population [23, 25].

The literature recognizes the limitations regarding the use of DDD in pediatric patients, so some authors believe that DOT is more appropriate for this population, particularly for low weight patients [17, 23, 40].

Regarding microbiological characteristics, the Brazilian study published in 2016 [3] also showed an increase in the occurrence *Candida* non*-albicans,* corresponding to 65.7% of the cases, although in isolation *C. albicans* still remains as the predominant species at 34.3%, followed by *C. parapsilosis* (24.1%), *C. tropicalis* (15.3%) and *C. glabrata* (10.2%). These results are similar to those of our study, where *C. albicans* the most isolated species corresponded 36.5% of the cases, followed by *C. parapsilosis* (22.6%), *C. glabrata* (21.7*%*) and *C. tropicalis* (11.3%), differing only in the rank order and percentages for *C. tropicalis* and *C. glabrata.* In the prior study carried out in our institution from 1997 to 2007, *C. albicans* was the most isolated species in blood cultures (44%), followed by *C. parapsilosis* (22%), *C. tropicalis* (15%) and *C. glabrata* (9%) [1]. In view of these data, over the years we can see the increase of *C.* non*-albicans* species in our institution as well, as reported in other studies [5, 41, 42].

We did not analyze the change in antifungal susceptibilities over the years in this study, but we could make a comparison with a prior study carried out in this institution from 1997 to 2007 whose susceptibility testing data are from mid-2002 to 2007 [1]. This showed that the resistance profile remained proportionally the same, with 100% of strains susceptible to amphotericin B and 91.4% (*n =* 96/105) of the strains susceptible to fluconazole, compared to 82.3% in the prior study (*n =* 42/51). Table 6 shows the susceptibility tests of *Candida* species isolates found in the first study conducted at our institution, data from mid-2002 to 2007 [1] and the findings in our study from 2012 to 2016. Among the resistant and intermediate species (susceptible dose-dependent) are *C. krusei* and *C. glabrata*, respectively. Until 2007, we only had caspofungin on formulary at our institution, and until then no susceptibility tests were found with intermediate results for this drug. However, in the current study, we have already seen the appearance of intermediate susceptibility to echinocandins, although it is a small percentage of strains, we can consider this as a warning sign to prompt increased monitoring of the use of these class of drugs, since the use of antifungal drugs affects the susceptibility of *Candida* species [43] and the selection of resistant strains may be responsible for failures in treatment [44].

**Table 6:** Comparison of microbiological data susceptibility tests between our study and Camargo et al. study [1]

Variables	*n*(%)
2002 a 2007^*^	
*Candida albicans* (*n =* 27)	
Amphotericin B (*n =* 27)	
Susceptible	27 (100.0)
Fluconazole (*n =* 27)	
Susceptible	27 (100.0)
*Candida* non-*albicans* (*n =* 51)	
Amphotericin B (*n =* 51)	
Susceptible	51 (100.0)
Fluconazole (*n =* 51)	
Susceptible	42 (82.3)
2012 a 2016^**^	
*Candida albicans* (*n =* 42)	
Amphotericin B (*n =* 40)	
Susceptible	40 (100.0)
Fluconazole (*n =* 41)	
Susceptible	41 (100.0)
Voriconazole (*n* = 41)	
Susceptible	41 (100.0)
Caspofungin (*n =* 1)	
Susceptible	1 (100.0)
Anidulafungin (*n =* 3)	
Susceptible	3 (100.0)
*Candida* non-*albicans* (*n =* 73)	
Amphotericin B (*n =* 72)	
Susceptible	72 (100.0)
Fluconazole (*n =* 64)	
Susceptible	55 (85.9)
Resistant	3 (4.7)
Intermediate	6 (9.4)
Voriconazole (*n* = 64)	
Susceptible	63 (98.4)
Intermediate	1 (1.6)
Caspofungin (*n* = 10)	
Susceptible	9 (90.0)
ntermediate	1 (10.0)
Anidulafungin (*n* = 11)	
Susceptible	8 (72.7)
Intermediate	3 (27.3)

Category variables presented by absolute and relative frequencies

^*^Data from Camargo TZS, et al. study [1]. ^**^Data from our current study

In a study carried out in Costa Rica [43], 19% of adult patients with candidemia did not receive antifungal drugs and these patients had a significantly higher risk of death within the next 30 days when compared to patients receiving antifungal drugs (OR: 2.9, 95% CI 1.8 to 4.6, *p* < 0.001). In our study, 13.9% of patients who had candidemia did not receive antifungal drugs; however, treatment with antifungals was associated with reduced mortality in multivariate analysis. In the Costa Rican study [45], 82% of candidemic patients started antifungal therapy more than 24 h after the blood culture was obtained. In our study the proportion was much smaller (41.7%) and no significant association was found between the timing of empiric therapy and mortality. In a European study, the proportion of patients receiving adequate antifungal therapy in the first 24 h after blood culture was high, and the delay in initiating antifungal therapy was not a risk factor for death [46].

In another study, which involved patients in septic shock, no differences were found in mortality regarding the timing of the beginning of antifungal therapy, but they identified a higher mortality in the group of patients where antifungal therapy was not adequate (p < 0.001) [12]. In our study, for most patients antifungal doses were in accordance with the institutional protocol but to our surprise the fluconazole dose was only adequate in 24.5% of the cases, with the majority being prescribed in suboptimal doses for the treatment of candidemia. The AmarCAND2 group study [47] also showed that the dose of fluconazole was not adequate in most patients. In view of these numbers, we can conclude that the pharmacist’s performance in the verification of the medical prescription by checking the doses and frequencies of the medications is very important, since treatment with inadequate doses may lead to unfavorable clinical outcomes.

In another study there was no correlation between the reduction of in-hospital mortality and the time of initiation of the therapy or initial choice of agents; instead they observed that risk factors, such as age, admission to the ICU or diabetes mellitus, have a strong impact on outcomes [13]. On the other hand, another study identified that mortality was lower when fluconazole therapy was started on the same day the culture was obtained [11]. We observed that there are still differences in the literature regarding whether the appropriate therapy actually impacts the risk of death, but it is clear that there are other factors that strongly influence the mortality related to candidemia, such as the severity of illness and the presence of comorbidities [13, 48].

An American study identified through univariate analysis that the predictors of increased mortality included the APACHE II severity of illness score, intensive care unit admission, corticosteroid treatment, and the use of mechanical ventilation [11]. In our study, independent predictors of mortality age, ICU length of stay, use of vasoactive drugs, chemotherapy, hemodialysis, and breakthrough candidemia*.*

The Eurobact study [44] identified mechanical ventilation and ICU admission as independent risk factors for death. These variables were not independent predictors in our study, but the length of ICU stay was an independent predictor of mortality.

Our study has some limitations primarily that it is single center and retrospective. The retrospective nature of the study made it impossible to identify the reason for the choice of specific antifungal drugs as initial therapy or the reason for the drug exchange during antifungal therapy, and also it was not possible to identify the number of cases where the antifungal was used as prophylaxis. To reduce bias, we chose to include only microbiological data from blood cultures to evaluate adequacy of antifungal therapy, since for a positive culture is highly predictive of infection and requires intervention, which is not characteristic of other sites, such as the urinary or respiratory tracts, where positivity may be due to colonization, which makes results difficult to interpret.

## Conclusion

The high utilization of antifungal drugs reinforces the need for ongoing monitoring, the implementation of stewardship interventions, and the key role of the pharmacist to increase rational use and thus improve patient safety.

## Abbreviations

AIC: Akaike information
AICU: Adult intensive care unit
APACHE II: Acute physiology and chronic health evaluation II
ATC: Anatomical therapeutic chemical
BMT: Bone marrow transplantation
CI: Confidence intervals
DDD: Defined daily dose
IDD: Individualized daily dose
DOT: Days of therapy
ICU: Intensive care unit
MSW: Medical and surgical wards
NICU: Neonatal intensive care unit
ONCO: Oncology
OR: Odds ratios
PDD: Prescribed daily dose
PED: Pediatrics
RDD: Recommended daily dose
SOT: Solid organ transplantation
WHO: World Health Organization
